# Two-Phase Equilibrium Conditions in Nanopores

**DOI:** 10.3390/nano10040608

**Published:** 2020-03-26

**Authors:** Michael T. Rauter, Olav Galteland, Máté Erdős, Othonas A. Moultos, Thijs J. H. Vlugt, Sondre K. Schnell, Dick Bedeaux, Signe Kjelstrup

**Affiliations:** 1 PoreLab, Department of Chemistry, Norwegian University of Science and Technology, NO-7491 Trondheim, Norway; olav.galteland@ntnu.no (O.G.); dick.bedeaux@ntnu.no (D.B.); signe.kjelstrup@ntnu.no (S.K.); 2 Engineering Thermodynamics, Process and Energy Department, Delft University of Technology, Leeghwaterstraat 39, 2628CB Delft, The Netherlands; m.erdos-2@tudelft.nl (M.E.); o.moultos@tudelft.nl (O.A.M.); T.J.H.Vlugt@tudelft.nl (T.J.H.V.); 3 Department of Materials Science and Engineering, Norwegian University of Science and Technology, NO-7491 Trondheim, Norway; sondre.k.schnell@ntnu.no

**Keywords:** pressure, confinement, equilibrium, thermodynamic, small-system, hills-thermodynamics, pore, nanopore, interface

## Abstract

It is known that thermodynamic properties of a system change upon confinement. To know how, is important for modelling of porous media. We propose to use Hill’s systematic thermodynamic analysis of confined systems to describe two-phase equilibrium in a nanopore. The integral pressure, as defined by the compression energy of a small volume, is then central. We show that the integral pressure is constant along a slit pore with a liquid and vapor in equilibrium, when Young and Young–Laplace’s laws apply. The integral pressure of a bulk fluid in a slit pore at mechanical equilibrium can be understood as the average tangential pressure inside the pore. The pressure at mechanical equilibrium, now named differential pressure, is the average of the trace of the mechanical pressure tensor divided by three as before. Using molecular dynamics simulations, we computed the integral and differential pressures, $\hat{p}$ and *p*, respectively, analysing the data with a growing-core methodology. The value of the bulk pressure was confirmed by Gibbs ensemble Monte Carlo simulations. The pressure difference times the volume, *V*, is the subdivision potential of Hill, $(p-\hat{p})V=\epsilon$. The combined simulation results confirm that the integral pressure is constant along the pore, and that ϵ/V scales with the inverse pore width. This scaling law will be useful for prediction of thermodynamic properties of confined systems in more complicated geometries.

## 1. Introduction

It is well known that thermodynamic properties of fluids significantly change when the fluid phases become confined [1,2]. Given the importance of porous media for applications in for example fuel cells [3], batteries [4], and membranes for drinking water production [5,6,7], it is essential to understand how properties change upon confinement. In particular, one would like to be able to predict how thermodynamic properties of the system change with size. We have earlier documented how the thermodynamic factors and the pressures of some systems scale with a characteristic inverse system size (surface area over volume) [8,9,10]. The aim of the present work is to continue this line of work and present a geometric scaling law for the pressure of a nanopore. This work can, in a general context, be seen as an extension of Hill’s thermodynamics of small systems [11]. The essential idea of Hill was to introduce a large ensemble of N small systems, enabling the use of standard thermodynamic tools for the ensemble. System properties were obtained by dividing the ensemble value by N. In this way, he was able to deal with the impact of shape- and size-variation of the small system, within the normal structure of thermodynamics. The dimensions of a nano-pore, which make molecular interactions at the pore surface important, can promote changes in phase transitions, surface adsorption, pore vapor pressure, and phase stability [2,12,13,14]. The literature offers, however, several definitions of the pressure inside a nano-porous material [15,16]. This situation calls for a robust definition of the representative elementary volume (REV) of the small system, to serve as a basis for a definition of pressure. Kjelstrup et al. [17] offered a definition of the REV-pressure based on the compression energy of the REV. Galteland et al. [18] showed, by evaluating this property for a single component in a nanoporous system, that the REV was not sufficiently described by the bulk fluid pressure. Also the integral pressure, $\hat{p}$, as defined by Hill was required to describe the degree of confinement in the REV. In Hill’s [11] terminology, one distinguishes between the integral pressure $\hat{p}$, and the differential pressure, *p*. Erdos et al. [10] provided an expression for the ratio of the driving forces for adsorption into a pore, by means of the integral and differential pressures. They expressed the ratio by the cross-correlation of the volume and the integral pressure.

Hill defined the difference of the two pressures times the volume by the subdivision potential $\epsilon =(p-\hat{p})V$ [11]. The integral pressure can differ significantly from the differential pressure in confined systems [10,18]. In the limit of a bulk fluid, they are the same, however. Galteland et al. studied pores between an fcc type-lattice of spheres [18], and found that the difference in the differential and integral pressure depended on the inverse radius of the spheres in the lattice. Here, we investigate a slit pore of nano-scale dimensions, with a constant pore slit-width ranging from 3 to 5 nm, and filled with a vapor and liquid phase of the same component. The purpose is to find thermodynamic and mechanical equilibrium conditions in terms of the pressures defined by Hill, and compare these to existing descriptions in terms of Young’s and Young–Laplace’s laws. The analysis leads to a new equilibrium condition, stated in terms of the integral pressure: This property is constant along the nano-sized pore. When this condition is combined with Youngs’ equation for the contact angles, one recovers the Young–Laplace equation.

For the geometry studied here, it is possible to derive a scaling law for the integral pressure. The pore shape is kept constant in the derivations, as shape is a variable in small system thermodynamics [19]. The new scaling law relates properties of one system size to another size, with the same shape. This is helpful in a situation where measurements are lacking. Hill extended the thermodynamic variable set, including an ensemble of the small system, thereby providing a new basis for introduction and examination of size effects in thermodynamics. Hill’s formulation of surface thermodynamics contain Gibbs’ method [20].

Molecular dynamics and Monte Carlo simulations [10,21] are excellent tools for equilibrium studies of confined systems. Two fluid phases will be simulated in a slit pore of varying width. Focus is directed to the equilibrium conditions, when single phases are in contact with each other and the wall. The choice of the system was motivated by the simple geometry, which makes it possible to test particular scaling laws for slit pores and cylindrical pores.

The structure of the paper is as follows: We first present the equations of a REV leading to the relation between the differential pressure *p* and the integral pressure $\hat{p}$. We next test the derived equations for slit pores of different sizes and different interactions between pore wall and fluid. The new growing-core methodology, brings out the difference between the differential and the integral pressure. Finally, results are discussed and put in perspective.

## 2. Theory

### 2.1. Thermodynamic Relations for Small Systems

Hill used the name “small system” for systems with properties that are not extensive in the system volume [11]. Such systems are frequently confined, i.e., bounded by the surroundings. To obtain a generalized thermodynamic description of small systems, and to deal with size and shape-dependencies, Hill [11] introduced an ensemble of N identical and independent replicas of the small system. Keeping the total entropy $S_{t}$, and particle number, $N_{t}$, constant, while increasing the number of small systems N (each with constant volume *V*) in the ensemble, an extended version of the Gibbs equation was obtained:

(1) $$dU_{t}=TdS_{t}-pNdV+\sum_{j}\mu_{j}dN_{j,t}-\hat{p}Vd\;N$$

which we call the Hill-Gibbs equation. Here, $U_{t}$ is the total internal energy of the ensemble, *T* is the temperature, *p* is the pressure, and $\mu_{j}$ is the chemical potential of component *j*. The new term, $-\hat{p}Vd\;N$, is the reversible work needed to change the total volume, by changing the number of small systems, keeping $S_{t}$, *V*, and $N_{t,j}$ constant.

Hill [11] distinguished between *p* and $\hat{p}$ by calling the properties the differential and integral pressure, respectively. This work aims to elucidate the difference of these variables for a small system which is confined to a pore, in the grand-canonical ensemble.

The average values in the grand-canonical ensemble for a single small system are

(2) $$U=\frac{U_{t}}{N},\;N_{j}=\frac{N_{j,t}}{N},\;S=\frac{S_{t}}{N}.$$

By introducing these equations into Equation (1), we obtain the usual Gibbs equation for dU, and what we call the Hill-Gibbs-Duhem’s equation.

(3) $$d\left(\hat{p}V\right)=SdT+\sum_{j}N_{j}d\mu_{j}+pdV$$

The equation reduces to the usual Gibbs-Duhem equation when $\hat{p}=p$. When *T* and $\mu_{j}$ are constant in Equation (3), we obtain a relation between the integral and differential pressure when the shape of the system is controlled [11]:

(4) $$p={\left(\frac{\partial \hat{p}V}{\partial V}\right)}_{T,\mu }=\hat{p}+V{\left(\frac{\partial \hat{p}}{\partial V}\right)}_{T,\mu }.$$

The expression shows that *p* and $\hat{p}$ differ for small systems, for which $\hat{p}$ depends on the volume. In a macroscopic system, with V→∞, *p* and $\hat{p}$ are the same. The relation clarifies the chosen names; *p* is obtained by differentiation.

The above equations will be applied to a two-phase system in a slit pore and a cylindrical pore. Inside the pore, the differential pressure of the liquid differ from that of the vapor phase. For the time being, we introduce the notation $p_{d}^{v}$ and $p_{d}^{l}$ for the differential pressure of the vapor and liquid, respectively, and $p_{d}^{f}$ to indicate either of the two. The difference of the differential and the integral pressure times the volume is equal to the subdivision potential [11], ϵ. By rearranging Equation (4), using $p_{d}^{f}=p$, we obtain:

(5) $$p-\hat{p}=\frac{\epsilon (T,V,\mu_{j})}{V}=V{\left(\frac{\partial \hat{p}}{\partial V}\right)}_{T,\mu_{j}}.$$

The subdivision potential is an additive thermodynamic property [11]. When such a property is divided by the small system volume, a scaling law can be found; of a similar type as we have found earlier [8,9]. The ratio ϵ/V is proportional to the the inverse characteristic size Ω/V of the system, where Ω is the surface area between the fluid and the solid [18]. In general terms,

(6) $$p-\hat{p}=\frac{\epsilon }{V}=\zeta^{\infty }+\zeta^{s}\left(\frac{\Omega }{V}\right)+\zeta^{se}{\left(\frac{\Omega }{V}\right)}^{2}+\ldots$$

The coefficients $\zeta^{\infty },\zeta^{s}$ and $\zeta^{se}$ do not depend on Ω/V, but are functions of *T* and $\mu_{j}$. As *p* and $\hat{p}$ are equal in the thermodynamic limit, it follows that the coefficient $\zeta^{\infty }=0$.

### 2.2. The Integral Pressure of a Representative Volume Element

To define the differential and integral pressures, and the conditions for thermodynamic and mechanical equilibrium between two phases within a pore, we need suitable REVs. In a porous medium with two fluid phases, we need more than one type of REV, one for each phase. A REV needs, as the name says, to be representative for all molecular interactions in the system [17]. It should be large enough to contain a statistically representative amount of the fluids and solids. The purpose of the REV is to define a volume element, to be used to define equilibrium, but also to define local equilibrium in a large system where fluid transports take place [17].

**Figure 1:**
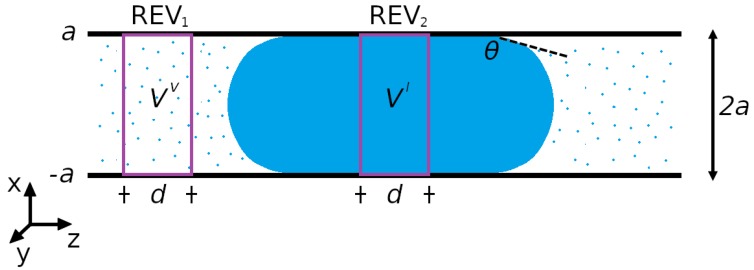
Slit pore of width 2a with a liquid droplet in the middle, which is in equilibrium with a vapor phase on both sides. The representative volume elements are REV${}_{1}$ (vapor of volume $V^{v}$) and REV${}_{2}$ (liquid of volume $V^{l}$), both with length *d*. The *x*-axis is located at the center of the pore.

A liquid droplet in equilibrium with its vapor inside a slit pore is an example of a two-phase confined fluid. The droplet system is illustrated in Figure 1. Various REVs are possible. Each REV need to cover the whole cross section of the pore. In the following we are concerned with two REVs, one in the vapor- and one in the liquid phase. None of them includes the surface between liquid and vapor. Two examples of REVs (REV${}_{1}$ and REV${}_{2}$) are illustrated in Figure 1. The *x*-axis is located at the center of the pore.

The integral pressure of an isothermal REV with constant chemical potentials is expressed in terms of the compression energy, $\hat{p}V$, which is defined by the grand potential, Y [11].

(7) $$\hat{p}V=-Y\equiv k_{B}T\ln\Xi$$

Here $k_{B}$ is the Boltzmann constant and Ξ the grand-canonical partition function. Similar to ϵ, also the compression energy is additive. It is the sum of contributions from all phases and interfaces present within the REV. The grand potential provides then the statistical mechanical link to particle behaviour, and is a tool for pressure computations (see below). The system is small in the sense of Hill, when the integral pressure deviate from the (bulk) fluid pressure.

The general expression for the compression energy for a REV${}_{n}$, with n=1,2 is then

(8) $${\hat{p}}_{{\mathrm{REV}}_{n}}V={\hat{p}}^{f}V^{f}-{\hat{\gamma }}^{fs}\Omega^{fs}$$

where *V* is the volume of the REV, $V^{f}$ is the volume of the fluid, $\Omega^{fs}$ is the surface area between the fluid and the wall, and ${\hat{\gamma }}^{fs}$ is the integral surface tension at the wall, or the surface tension of the small system. In the present case, the two REVs are completely filled with a fluid; a vapor (REV${}_{1}$) or a liquid (REV${}_{2}$). The REV volume in both cases obeys $V=V^{f}=V^{v}=V^{l}=2adw$, where *a* is the slit pore half width, *d* the REV length and *w* the width of the REV. Superscripts *v* and *l* refer again to the vapor and the liquid. The surface area between fluid and solid is $\Omega^{fs}=2dw$. The surface tension between fluid and pore surface are ${\hat{\gamma }}^{ls}$ and ${\hat{\gamma }}^{vs}$, respectively, where the hat indicate that the system may be small. In that case, the surface tension is a function of the contact area/system geometry.

For sufficiently large values of *a*, the fluid in the center of the pore has macroscopic bulk properties. In that case; the integral pressure in the vapor ${\hat{p}}^{v}$ and the liquid ${\hat{p}}^{l}$ are independent of $V^{v}$ or $V^{l}$, respectively, and

(9) $${\hat{p}}^{v}=p^{v},\;{\hat{p}}^{l}=p^{l}.$$

Here, $p^{l}$ and $p^{v}$ are bulk pressures of the liquid and vapor, respectively.

In a slit pore, the wall surface is not curved. When the distance between the walls (2a) is sufficiently large, the fluid–solid surface tension show macroscopic behaviour, and:

(10) $${\hat{\gamma }}^{vs}=\gamma^{vs},\;{\hat{\gamma }}^{ls}=\gamma^{ls}.$$

By introducing the expressions, Equations (9) and (10), the volume and the fluid–solid surface area into Equation (8), we obtain expressions for the integral pressure of the liquid and vapor REVs:

(11) $${\hat{p}}_{{\mathrm{REV}}_{2}}=p^{l}-\frac{\gamma^{ls}}{a},\;{\hat{p}}_{{\mathrm{REV}}_{1}}=p^{v}-\frac{\gamma^{vs}}{a}.$$

Suppose now that integral pressure is constant everywhere at equilibrium, or that:

(12) $${\hat{p}}_{{\mathrm{REV}}_{2}}={\hat{p}}_{{\mathrm{REV}}_{1}}.$$

This means we can write:

(13) $$p^{l}=p^{v}+\frac{\gamma^{ls}-\gamma^{vs}}{a}$$

Young’s law derives from a force balance at phase equilibrium:

(14) $$\gamma^{ls}=\gamma^{vs}+\gamma^{lv}\cos\theta ,$$

Here $\gamma^{lv}$ is the liquid-vapor surface tension. We introduce this law in the equation above and obtain:

(15) $$p^{l}=p^{v}+\frac{\gamma^{lv}}{a}\cos\theta =p^{v}+\frac{\gamma^{lv}}{R}.$$

This is Young–Laplace’s equation for a slit pore. Young–Laplace’s equation has been verified in the experimental literature numerous times [22,23]. The radius of curvature of the liquid-vapor surface, R=a/cosθ, was used, where θ is the contact angle at the pore wall, see Figure 1. We have found this equation without examining the liquid-vapor interface per se.

Equation (12) is thus equivalent to validity of the laws of Young and Young–Laplace. These familiar laws apply to thermodynamic and mechanical equilibrium, so we expect the equality to do the same. In this sense we have derived a new way to express phase equilibrium in nanopores.

Consider again a state of equilibrium where the integral pressure is constant. The integral pressures of the fluids are shown by Equation (11). For each fluid, the difference of the differential and integral pressure for the slit pore follows from Equation (4):

(16) $$p-\hat{p}=a^{3}{\left(\frac{\partial \hat{p}}{\partial a^{3}}\right)}_{T,\mu }=\frac{\gamma^{fs}\left(\alpha \right)}{3a}.$$

where we have indicated that the surface tension depends on a parameter α which characterizes the molecular interactions of the fluid and the wall. The shape is a variable in small system thermodynamics, and remains constant when we determine the derivative. To keep the shape constant, means to take the isomorphic derivative of the integral pressure in Equation (16).

A change from the slit pore to a cylindrical pore, will change the proportionality factor in a concrete way. The ratio of the area over the volume in the REV of a cylindrical pore is double the value in a slit pore. The ratio of the slope in a cylindrical pore over that from a slit pore is therefore equal to 2. The corresponding scaling law for a cylindrical pore becomes:

(17) $$p-\hat{p}=a^{3}{\left(\frac{\partial \hat{p}}{\partial a^{3}}\right)}_{T,\mu }=\frac{2\gamma^{fs}\left(\alpha \right)}{3a}.$$

By changing the geometry of the pore only, keeping the fluid wall interactions constant, we predict that the slope of pressure difference vs the surface tension will change by a factor 2.

For large systems (when a→∞), the difference between *p* and $\hat{p}$ disappears, leading to $p=\hat{p}=p^{f}$ in the REV. For small systems, however, the difference between *p* and $\hat{p}$ is described by the ratio between fluid–solid surface tension and the half channel width ($\gamma^{fs}/a$) divided by 3. We have thus found that the pressure difference follows a scaling law, similar to the one proposed by the Small System Method [8,9], and given in Equation (6).

Equation (16) can be tested by molecular dynamics simulations. In such a test, one can identify independently all terms in the equation, and confirm that the condition ${\hat{p}}_{{REV}_{1}}={\hat{p}}_{{REV}_{2}}$ holds. Essential in the calculation is that *p* and $\hat{p}$ refer to the same volume.

These theoretical results demonstrate why it may pay to take the effort to invoke the perhaps more complicated thermodynamic scheme of Hill. Young and Young–Laplaces’ laws describe the same physical phenomenon as Equations (16) and (17). Hill’s theory shows how the two Equations (16) and (17) are linked to each other and to new variables (e.g., ϵ), in manners which can be tested.

### 2.3. A Mechanical Interpretation of the Pressures

The above derivation showed that the condition of constant integral pressure at equilibrium is required by Young’s and Young–Laplace’s laws. The integral and differential pressures are therefore also related to the mechanical pressure tensor. The mechanical pressure tensor will from now be denoted with upper case *P*, while the thermodynamic pressures are denoted as before, with lower case *p*. A complete set of relations between *P* and *p* is provided in the Appendix A. Here, we present the main relations.

Consider again the liquid droplet in a slit pore, as shown in Figure 1. The slit pore wall is parallel to the yz-plane. The pressure tensor component acting normal to the wall is the xx-component, $P_{N}=P_{xx}$ (cf. the Appendix A). There are two tangential components of the pressure tensor, the yy-component and the zz-component, which are equal in equilibrium. The tangential pressure tensor component is the average of the two, $P_{T}=\frac{1}{2}\left(P_{yy}+P_{zz}\right)$. The surface tension is minus the excess of the tangential pressure tensor [24],

(18) $$\gamma^{fs}=\frac{1}{2}\int_{-a}^{a}\left(P_{N}\left(x\right)-P_{T}\left(x\right)\right)dx.$$

The factor of one half accounts for the fact that the integral is for two fluid–solid surfaces. By introducing the fluid–solid surface tension into the equation for the integral pressure of a single fluid phase, cf. Equation (11), we obtain

(19) $$\hat{p}=p^{f}-\frac{1}{2a}\int_{-a}^{a}\left(P_{N}\left(x\right)-P_{T}\left(x\right)\right)dx,$$

where superscript *f* again refers to either the liquid or vapor. The normal component of the mechanical pressure tensor is constant through flat surfaces at mechanical equilibrium. The integral of the normal component divided by 2a is therefore equal to $P_{N}$. We assume that the normal component is equal to the differential pressure of the bulk fluid, $p^{f}=P_{N}$. Below, the assumption is shown to hold.

The differential pressure of the bulk fluid and the integral over the normal component of the pressure cancel each other, and the integral pressure is equal to the average tangential component,

(20) $$\hat{p}=\frac{1}{2a}\int_{-a}^{a}P_{T}\left(x\right)dx=\langle P_{T}\rangle .$$

This equation shows that the integral pressure of a bulk fluid in a slit pore at mechanical equilibrium can be understood as the average tangential pressure.

The differential pressure can also be related to the mechanical pressure tensor. The difference between the differential and integral pressures is equal to the fluid–solid surface tension divided by 3a. By introducing Equation (18) for the fluid–solid surface tension, we obtain

(21) $$p-\hat{p}=\frac{1}{6a}\int_{-a}^{a}\left(P_{N}\left(x\right)-P_{T}\left(x\right)\right)dx$$

By reorganizing and inserting the average of the tangential pressure for the integral pressure,

(22) $$p=\frac{1}{3}\left(\langle P_{N}\rangle +2\langle P_{T}\rangle \right)=\frac{1}{3}\mathrm{Tr}\langle P\rangle$$

The differential pressure is equal to the average of the trace of the mechanical pressure tensor divided by 3.

## 3. Simulations

### 3.1. Molecular Dynamic Simulations

#### 3.1.1. System

**Figure 2:**
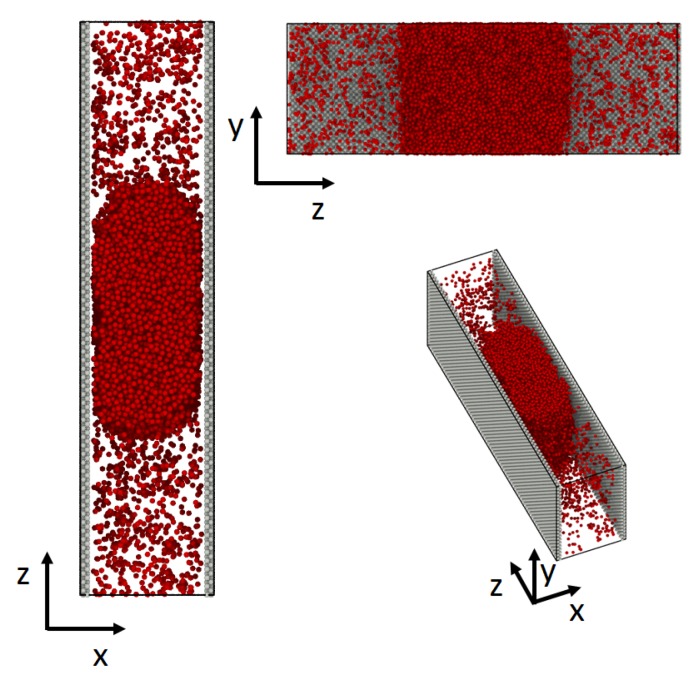
System used in the simulation. The droplet in the middle of the slit pore is surrounded by a vapor phase on both sides.

Simulations were carried out for a droplet in a slit pore. The droplet in the middle of the pore was surrounded by its vapor phase on both sides (see Figure 2). The simulation box, with the side lengths $L_{x}=L_{y}\neq L_{z}$ had periodic boundary conditions in all directions.

The simulation box was divided into *n* rectangular layers along the *z*-axis with a layer thickness of $\Delta z=L_{z}/n$. Both the pore-wall (grey) and the fluid (red) consisted of Lennard-Jones/spline particles [25]. The particles of the wall were immobilized, such that the particles were able to interact with the fluid particles, but not move. Due to periodic boundary conditions, the wall thickness was $t=3.5\sigma_{0}$ and had a density of $\rho_{wall}=1.05$. The fluid inside the pore was initialized in a way that the overall density inside the pore was $\rho_{fluid}=0.27$, thus in the two phase regime [25]. The averaged density of the bulk liquid was $\rho_{liquid}=0.75\pm 0.0015$ and the one of the vapor $\rho_{vapor}=0.03\pm 0.0025$.

The simulations were carried out using LAMMPS [26] in the canonical (NVT) ensemble at a temperature of $T^{*}$ = 0.70. The Nosé-Hoover thermostat [27] was used to keep the temperature constant. We used Lennard-Jones reduced units, which are indicated by superscript * [28]. The system was simulated for four slit widths, *a* = 8.7$\sigma_{0}$, 10.45$\sigma_{0}$, 12.2$\sigma_{0}$ and 13.7$\sigma_{0}$. The half channel widths were chosen so that both, the pressure of the fluid in the center, $p^{f}$, as well as the fluid–solid surface tension, $\gamma^{fs}$, are independent of the distance *a*, i.e., they show macroscopic bulk properties. Furthermore, the slit widths are large enough to avoid the effect of the disjoining pressure, as discussed in [13].

#### 3.1.2. Particle Interaction Potential

The interaction between particles of type *i* and *j* was defined by the Lennard-Jones/spline potential [25,28],

(23) $$u_{ij}\left(r\right)=\left\{\begin{array}{cc} 4\varepsilon_{ij}\left[{\left(\frac{\sigma_{ij}}{r}\right)}^{12}-\alpha_{ij}{\left(\frac{\sigma_{ij}}{r}\right)}^{6}\right] & \mathrm{if}r<r_{s,ij}\\ a_{ij}{\left(r-r_{c,ij}\right)}^{2}+b_{ij}{\left(r-r_{c,ij}\right)}^{3} & \mathrm{if}r_{s,ij}<r<r_{c,ij}\\ 0 & \mathrm{if}r>r_{c,ij} \end{array}\right.$$

where *r* is the distance between the particles. The interaction parameters, $\varepsilon_{ij}$ and $\sigma_{ij}$, were set to 1 for all particle pairs (Lennard-Jones units). The interaction parameter $\alpha_{ij}$ is used to control the attractive interaction between the wall and the fluid, where we used $\alpha_{sf}$ = 0.05, 0.15 and 0.25. For wall-wall and fluid-fluid interactions the interaction parameter was set to $\alpha_{ss}=\alpha_{ff}=1$. All $\alpha_{sf}$ values represent a strong, repulsive interaction between wall and fluid, thus a non-wetting behaviour. The parameters $a_{ij}$, $b_{ij}$, $r_{c,ij}$ and $r_{s,ij}$ were determined such that the potential and its derivative are continuous functions at $r_{s,ij}=1.24\sigma$ and $r_{c,ij}=1.74\sigma$ [25].

#### 3.1.3. Computation of REV Pressures and Wall-Fluid Surface Tension

The mechanical pressures in any layer *l* or combination of layers, or in a REV (core plus all layers included), were computed following Kirkwood [24]:

(24) $$P_{\beta \kappa }=\frac{1}{3V}\sum_{i\in l}m_{i}\left(v_{i,\beta }-v_{m,\beta }\right)\left(v_{i,\kappa }-v_{m,\kappa }\right)-\frac{1}{6V}\sum_{i\in l}\sum_{j=1}^{N}\left(r_{ij,\beta }f_{ij,\kappa }\right).$$

The subscripts β and κ denote the Cartesian coordinates, *x*, *y* and *z*, while *V* is the volume of the layer, $m_{i}$ is the mass of particle *i*, $v_{i,\beta }$ is the velocity of fluid particle *i* in the β-direction, and $v_{m,\beta }$ is the average velocity in the β-direction. This velocity is zero in our equilibrium simulations. The properties $r_{ij,\beta }$ and $f_{ij,\beta }$ are, respectively, the distance and the force between particle *i* and *j* in the β-direction. The first term is the kinetic energy contribution from the particles, and the second term arise from pairwise interactions. Because of isotropy, the tensor is diagonal (see Appendix A).

Half the value of the pairwise energy contribution is assigned to the layer that contains particle *i*, while the other half is assigned to the layer that contains particle *j*. A more precise distribution of the contributions is available [29,30,31,32], but the present method has sufficiently small errors for the volumes used.

**Figure 3:**
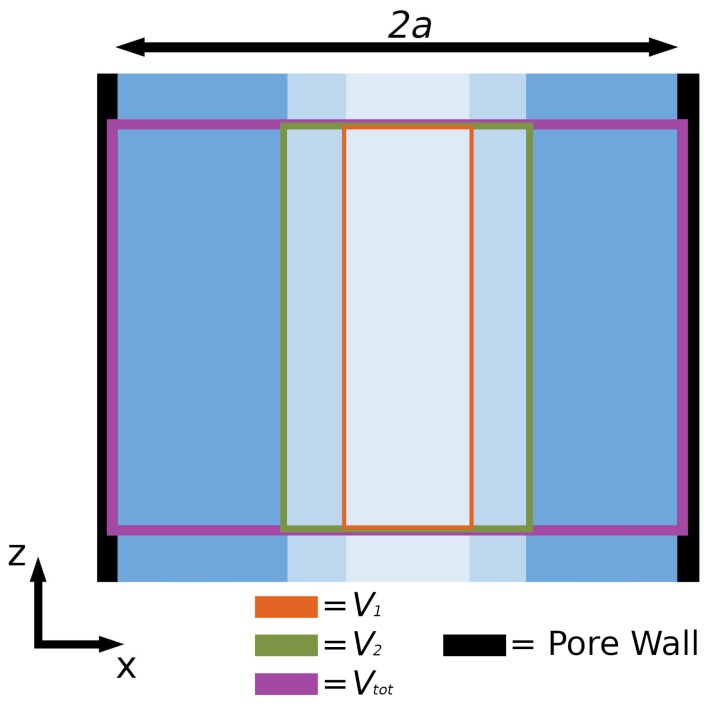
The core layer of the slit pore (lightest color) and subsequent layers (progressively darker color) used in the calculation of the pressure tensor of the fluid. $V_{1}$ and $V_{2}$ are the volumes of the core and the core plus one layer, respectively. $V_{tot}$ is the total volume, covering all interactions with the pore wall.

The pressure of a fluid volume element was calculated, starting with the core of the pore (lightest color in Figure 3), which has volume $V_{1}$. Volume was gradually added to the core, including in the end also the wall (black color in Figure 3). In this manner, the volume of the core was growing, layer by layer, until in the end s−1 layers were added to the central core. The pressure was computed for the core ($V_{1}$), for the core plus one subsequent layer ($V_{2}$), and for the total volume ($V_{\mathrm{tot}}$). The total volume, $V_{\mathrm{tot}}$, is equal to the volume *V* of the REV (see Figure 1). Thus, the pressure was computed in growing pore core -volumes until the volume was large enough to cover the whole width (2a) of the pore. This new growing-core methodology enabled an calculation of the pressure in the center of the pore (the core) and the averaged pressure of the whole cross section of the pore, including all interactions with the pore wall. When all layers plus the wall inside the pore were included ($V_{\mathrm{tot}}$), we obtained $\hat{p}$ of the REV. As long as there is no impact of the wall, (the core plus layers is sufficiently far away from the wall) we observed the bulk fluid pressure, $p^{f}$. This procedure was applied to two REVs, one in the liquid and one in the vapor phase.

The fluid–solid surface tension was computed as the excess of the tangential pressure tensor component [24] from Equation (18).

The extrapolated normal component $P_{N}$ was taken to be equal to the pressure in core, where the fluid–solid surface has no effect on the pressure. The tangential component of the pressure was calculated using $P_{T}=\frac{1}{2}\left(P_{yy}+P_{zz}\right)$. The value was obtained using a low resolution in *x*-direction and by averaging over multiple chunks of the liquid phase, along the pore in the *z*-direction.

### 3.2. Gibbs Ensemble Monte Carlo

In the derivation of Equation (20), the pressure tensor normal to the slit pore wall, $P_{N}$, is assumed to be equal to the bulk pressure $p^{f}$. This assumption was tested using Gibbs ensemble Monte Carlo simulations for two values of $p^{f}$ (0.2 and 6.0 reduced units). The method is the same as reported recently by Erdős et al. [10]. In the simulations, two simulation boxes were defined. Simulation box 1 represents a reservoir of a bulk fluid with periodic boundary conditions applied in all directions. The fluid particles interact with the shifted and truncated Lennard-Jones potential with σ=1, ϵ=1 and cut-off $r_{c}=2.5$. The pressure, $p^{f}$, and temperature, *T*, of the fluid in simulation box 1 are imposed, while the number of particles, $N_{1}$, and the volume of the box can fluctuate. Simulation box 2 was a slit pore with fixed volume and temperature. The size of the slit pore was defined by the distance between the two parallel planes, 2a. In simulation box 2, periodic boundary conditions were applied in the directions parallel to the planes. The total number of particles, $N_{1}+N_{2}$, was fixed in the simulations. Simulation box 1 and 2 can exchange particles, which ensured that the two boxes were in chemical equilibrium. The pressure of the fluid in simulation box 2 was equal to the differential pressure in equilibrium with the pressure imposed in the reservoir (box 1), $p^{f}$. A more detailed description of the simulation setup is given by Erdős et al. [10]. The high and low pressures were computed for varying pore sizes *a*.

## 4. Results and Discussion

**Figure 4:**
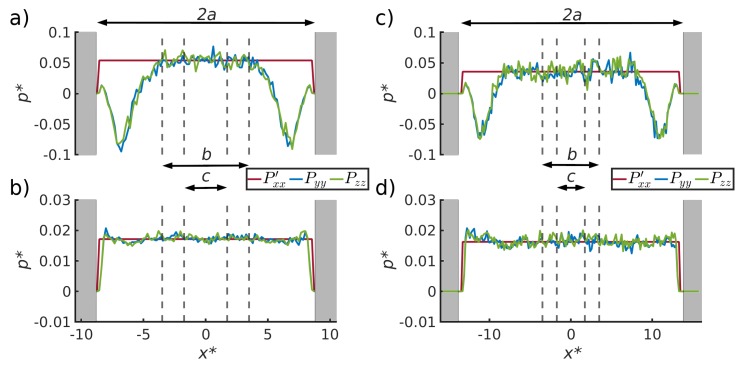
Pressure variation of the three pressure components normal to the surface shown for (**a**) the liquid phase with pore width 17.4$\sigma_{0}$, (**b**) the vapor phase with pore width 17.4$\sigma_{0}$, (**c**) the liquid phase with pore width 27.4$\sigma_{0}$ and (**d**) the vapor phase with pore width 27.4$\sigma_{0}$, for $\alpha_{sf}$ = 0.15. The normal component of the pressure, $P_{xx}^{\prime }$, is extrapolated from the center of the slit pore.

**Figure 5:**
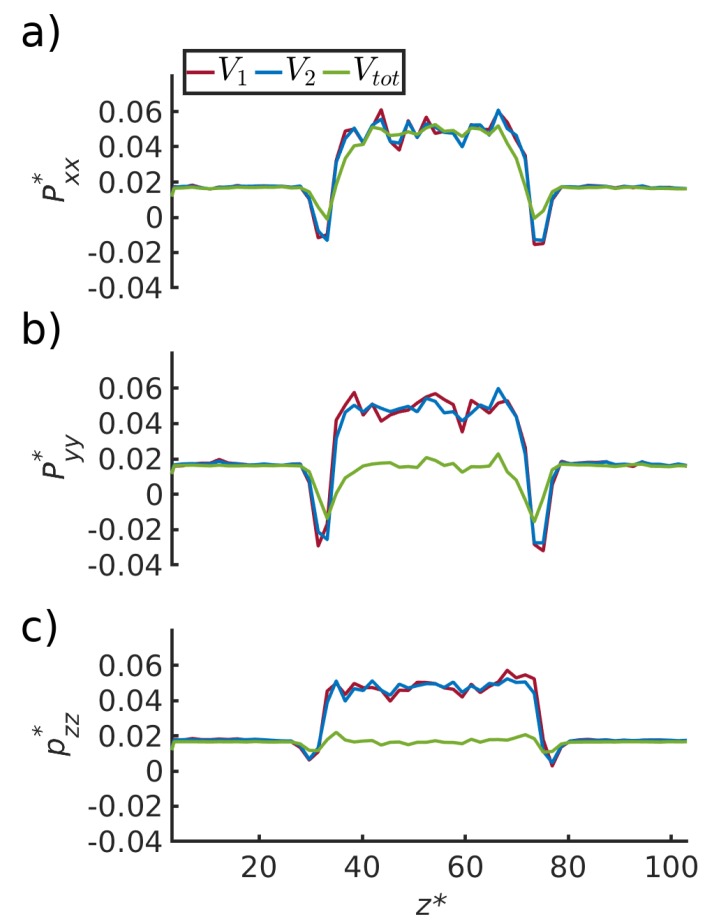
Pressure components (**a**) $P_{xx}$, (**b**) $P_{yy}$ and (**c**) $P_{zz}$, as a function of the *z*- direction for three regions defined using the growing-core methodology. The liquid droplet is in the center of the box, from $z=30\sigma_{0}$ to $z=75\sigma_{0}$. There is a vapor phase on both sides of the liquid droplet.

**Figure 6:**
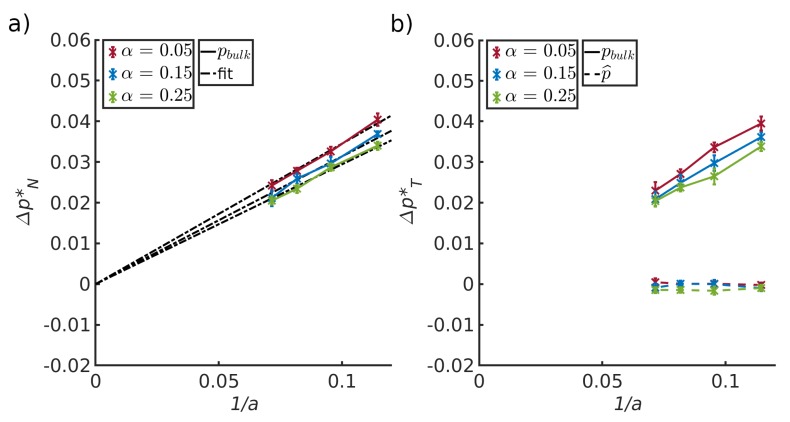
Difference between (**a**) the normal components of the pressure of the bulk liquid and vapor and (**b**) the difference in the tangential component of the pressure of the bulk liquid and vapor, for three different solid-fluid interactions (α) plotted as a function of the inverse half pore width. The bulk pressure, $p^{f}$ and the integral pressure are represented by solid and dashed lines, respectively.

**Figure 7:**
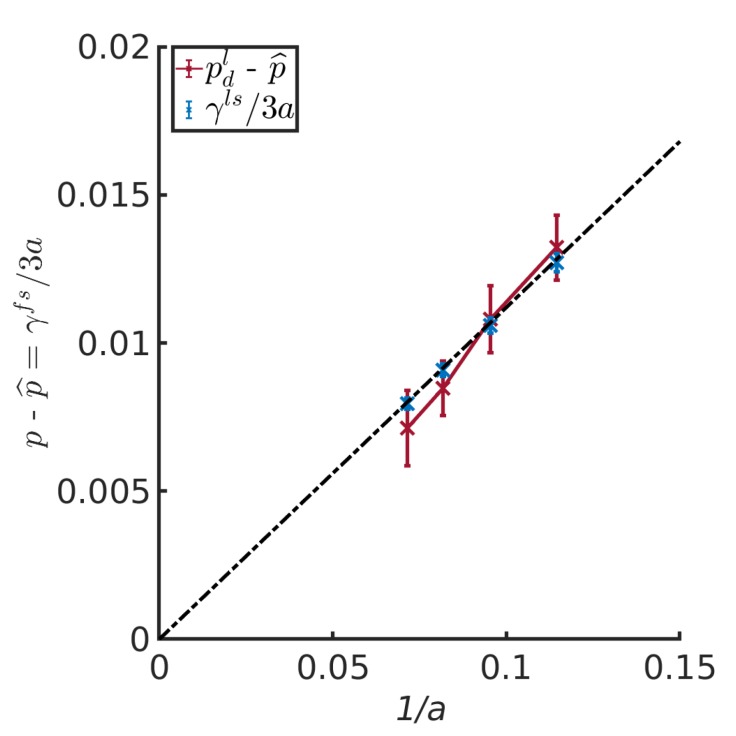
Difference between *p* and $\hat{p}$ for the liquid $REV_{2}$ as a function of the inverse half channel width for α=0.15..

The results for the independent computations of the pressure of a bulk fluid and corresponding pressure in the pore, using Gibbs ensemble Monte Carlo simulations, are shown in Table 1 in Section 4.1. The results of the molecular dynamics simulations are shown in Figure 4, Figure 5, Figure 6 and Figure 7 in Section 4.2, Section 4.3, Section 4.4 and Section 4.5. The figures show the pressure variation from the center of the slit to the wall, in the direction perpendicular to the wall (Figure 4) and parallel to the wall (Figure 5). The next figures confirm, as we shall see, that we can use a constant $\hat{p}$ to define equilibrium, and that a small system scaling law applies.

### 4.1. Pressure Component Normal to the Pore Wall

The normal component of the pressure tensor in simulation box 2 and bulk pressure in simulation box 1 are shown in Table 1.

The pressure tensor normal to the pore wall in simulation box 2 is according to Table 1 equal to the bulk pressure in simulation box 1 within the accuracy of the simulation. The results show that this assumption is correct. Therefore, the integral pressure can be understood as the average value of the pressure tensor components tangential to the pore wall, $\hat{p}=\langle P_{T}\rangle$.

### 4.2. Pressure Variation in the Direction Normal to the Pore Wall

The variation in the pressure component normal to the surface walls is shown for the liquid (top) and the vapor phases (bottom) in Figure 4, for pore widths, $2a=17.4\sigma_{0}$ (left) and $2a=27.4\sigma_{0}$ (right), using $\alpha_{sf}$ = 0.15 (repelling interactions). The horizontal straight line in all sub-figures is the xx-component extrapolated to the walls. We know there is bulk fluid in the core, as the pressures in $V_{1}$ and $V_{2}$ are the same, so this component is equal to the bulk pressure. For the other curves in the figure, the tangential components of the pressures (the yy- and zz-components) fall on top of each other. The distances 2a, *b* and *c* between the 4 dashed lines and the pore wall (grey) in the figure represent three fluid volumes, of increasing size ($V_{1},V_{2}$ and $V_{\mathrm{tot}}$). These are the core volume (width *c*, volume $V_{1}$), the core plus layer 1 (width *b*, volume $V_{2}$) and the whole volume (width 2a, volume $V_{\mathrm{tot}}$). The distance 2a is the width of the channel.

In the liquid phase (top), the tangential pressure component becomes negative close to the surface. This variation was used to compute the surface tension between solid and liquid from Equation (18). The value was found, within the accuracy of the calculation, to be independent of the distance between the two surface walls, see Table 2.

For the vapor phase, there is no dip in the pressure tensor into the negative regime. In the vapor, not only the extrapolated normal pressure component, $P_{xx}^{\prime }$, but also the two tangential components, $P_{yy}$ and $P_{zz}$, are constant and independent of the distance between the two surfaces. Therefore, the surface tension between the vapor and the wall is negligible.

The pressure in the pore core depends on the distance between the surface walls, i.e., it is larger in the pore with a smaller distance (left) than in the one with a larger distance (right). The bulk fluid pressures are related to the curvature of the liquid-vapor meniscus by Young–Laplace’s law, which depends on the slit pore width 2a.

### 4.3. Pressure Variation along the Pore

The pressure tensor components were computed for three volumes of increasing size ($V_{1},V_{2}$ and $V_{\mathrm{tot}}$) using the growing-core methodology. $V_{1}$ and $V_{2}$ are subvolumes in the center of the REVs and $V_{\mathrm{tot}}$ is the volume covering the whole REV, i.e., $V_{\mathrm{tot}}$ = $V^{l}$ = $V^{v}$ (see Figure 1 and Figure 3). In Figure 5, the pressure components are plotted as a function of the *z*-direction. The *z*-direction is parallel to the pore wall and passes through the vapor and the liquid droplet.

The volumes $V_{1}$ and $V_{2}$ contain bulk fluid, and are not influenced by the pore wall. The pressure components of the two volumes in the center of the box are equal. It was shown (Table 1) that the pressure was that of a bulk fluid in equilibrium with the pore. Therefore, the liquid bulk pressure is $p^{l}$. The pressures are equal to the vapor bulk pressure, $p^{v}$, on both sides of the liquid droplet.

The volume $V_{\mathrm{tot}}$ includes the fluid and its interface with the pore wall. The pressure component normal to the fluid wall is still equal to the fluid pressure, $P_{xx}=p^{f}$. The pressure components tangential to the fluid wall in the bulk liquid and bulk vapor, are equal to the integral pressure, $\hat{p}=\langle P_{T}\rangle$. The integral pressure is the same in the liquid and vapor phases, in agreement with Equation (20).

### 4.4. Pressure Differences Across the Liquid-Vapor Interface

Figure 6 (left) shows the difference between the normal components of the bulk pressures in the liquid and vapor. The pressure difference is shown as a function of the inverse half channel width, *a*, for three values of the interaction parameter α, all values representing wall-fluid repulsive forces. Figure 6 (right) shows the difference between the liquid and vapor for the tangential components of the pressures, i.e., the integral pressure $\hat{p}$ (dashed lines) and the bulk pressure $=p_{}^{f}$ (solid lines). Results are shown as a function of the inverse half channel width between the two pore walls, 1/a, and three fluid–solid interactions parameters α. These plots allow us to test Equations (10) and (12).

We first observe that the integral pressure differences for the two fluids are zero, meaning that the integral pressure is constant along the pore, see Figure 6 right bottom. The results support Equation (12). In the bulk phases, the tangential and normal components are the same. This confirms the soundness of the calculation procedures.

Both figures show that the difference in bulk pressure components depends on the distance between the two pore walls and the interaction parameter α. This is a consequence of a corresponding decrease of the liquid-vapor curvature. The uncertainties in the results, did not allow a determination of θ. We have therefore not computed the liquid-vapor surface tension, to confirm directly the law Young–Laplace. However, results of the two REVs combined, imply the validity of this law. The liquid–solid and vapor–solid surface tensions, computed with Equation (18), are given in Table 2 for the three α-values. The surface tension changes slightly with α.

Within the accuracy of the calculation, it was possible to estimate the slope of the three data sets. Lines to guide the eye were fitted through zero in the left-hand side figure, following Young–Laplace or the scaling law. The values of the slopes were 0.35/0.31/0.29, for α= 0.05/0.15/0.25, respectively. The slope is equal to $\gamma^{lv}\cos\theta$ which is approximately equal to $\gamma^{ls}$ since $\gamma^{vs}\approx 0$ (see Equation (14) and Table 2).

The slopes depended on the interaction parameter α. The value increased for decreasing values of α. This is expected: The more repelling the surface is, the higher is the surface tension. The slopes are in agreement with the liquid-solid surface tensions given in Table 2.

### 4.5. The Small System Scaling Law and the Subdivison Potential

Following the definition of Hill, the difference between the differential pressure, *p*, and the integral pressure, $\hat{p}$, was given by Equation (16), or $\epsilon /V=\gamma^{fs}/\left(3a\right)$. The relation is an example of a small system scaling law, cf. Equation (6).

To investigate this further, the differential pressure, *p*, of the liquid REV minus the integral pressure $\hat{p}$ of the same was plotted in Figure 7 as a function of the inverse half channel width for α=0.15. The difference between *p* and $\hat{p}$ was proportional to 1/a, and the slope was as predicted by the liquid/solid surface tension from Table 2 divided by the half channel width, *a*, and a factor of 3. The line goes through zero, when 1/*a* becomes zero. This is the thermodynamic limit value.

Erdos et al. [10] reported slopes for cylinder pores in addition to slit pores. The ratio of slopes between slit and cylinder pore was 0.52–0.58. From Equations (16) and (17), we obtain the ratio 0.50, when the surface tension is constant. The surface tension is a function of the interaction parameter, as we have seen. The surface tension may also depend on curvature. It follows that results from two pores with different geometries, having the same α, and a surface tension which is independent of curvature, will fall on top of each other.

The scaling law, that relate the integral and differential pressure of a REV, offer a new procedure for calculation of surface tensions. This defined dependence on the inverse characteristic length, may help distinguish between various other types of dependencies (curvature).

## 5. Conclusions

In this work we have confirmed, using molecular dynamics simulations, that the integral pressure in a nano-confined single fluid in two phases is constant along a pore. This supports and extends earlier investigations of Galteland et al. [18] for a single fluid between spheres arranged in a fcc lattice. The results imply, that Young’s and Young–Laplace equations apply, equations which have been validated experimentally numerous times. The integral pressure in mechanical equilibrium can be understood as the average tangential component of the pressure. It is this component that is constant through a pore at equilibrium. The differential pressure at mechanical equilibrium can be understood as the average of the trace of the mechanical pressure tensor divided by 3.

The integral pressure, introduced by Hill, is relevant for descriptions of nano-pores. Using this, we have developed a small system scaling law for nano-pores. Equation (6) is valid for a slit pore and a cylindrical pore. The findings allowed us to speculate on wider applications of the scaling law. One may imagine using the law for more complex geometries, where higher order terms are relevant (line tension contributions). Once the subdivision potential is known, other thermodynamic quantities, like the enthalpy-hat, can also be computed for the confined system [33]. This opens up new ways to study confined systems.

## Figures and Tables

**Table 1:** The pressure tensor normal to the slit pore wall, $P_{N}$, and the bulk pressure $p^{f}$ with varying pore sizes *a*. Two total pressures are studied for various pore sizes.

Pore Size $a/\sigma_{0}$	Normal Pressure, $P_{N}\sigma_{0}^{3}/\varepsilon_{0}$	Bulk Fluid Pressure, $p^{f}\sigma_{0}^{3}/\varepsilon_{0}$
5	6.178±0.005	6.023±0.005
6	6.017±0.005	6.011±0.005
9	6.014±0.005	5.995±0.006
12	6.011±0.005	5.995±0.003
15	6.029±0.004	5.995±0.004
21	6.011±0.004	6.000±0.003
27	5.997±0.004	6.002±0.003
35	5.998±0.004	5.997±0.003
5	0.2011±0.0009	0.2002±0.0002
6	0.1988±0.0009	0.2005±0.0002
9	0.2007±0.0008	0.2000±0.0002
12	0.2020±0.0007	0.2002±0.0002
15	0.1980±0.0007	0.1997±0.0002
21	0.1993±0.0006	0.1997±0.0001
27	0.2005±0.0006	0.2000±0.0001
35	0.2000±0.0005	0.2001±0.0001

**Table 2:** Surface tensions for the liquid/solid and vapor/solid interfaces. Values are presented for three interaction parameters α in the Lennard-Jones potential used in the computations. The surface tensions were computed via the excess of the tangential pressure tensor (see Equation (18)).

α	$\gamma^{ls}$	$\gamma^{vs}$
0.05	0.36 ± 0.02	0.0067 ± 0.0026
0.15	0.33 ± 0.01	0.005 ± 0.0035
0.25	0.31 ± 0.01	0.004 ± 0.0045

## Appendix A

In an isotropic system, the differential pressure *p* is normally defined by the trace of the pressure tensor [34]:

(A1) $$p=\frac{P_{xx}+P_{yy}+P_{zz}}{3}$$

where $P_{ii}$ is the pressure component in the direction ii. Also the integral pressure can be understood in terms of these pressure components. The integral pressure, $\hat{p}$, in the present slit pore was expressed by Equation (20):

(A2) $$\hat{p}=\frac{1}{2a}\int_{-a}^{a}P_{T}dx=\langle P_{T}\rangle =\frac{P_{yy}+P_{zz}}{2}$$

where $P_{yy}$ and $P_{zz}$ are the pressure components in tangential direction, and *x* is the direction normal to the surface. The difference between *p* and $\hat{p}$ can then be written as

(A3) $$p-\hat{p}=\frac{P_{xx}+P_{yy}+P_{zz}}{3}-\frac{P_{yy}+P_{zz}}{2}$$

which results in

(A4) $$p-\hat{p}=\frac{P_{xx}}{3}-\frac{P_{yy}+P_{zz}}{6}$$

By using the definition for the tangential pressure from Equation (A2), we obtain

(A5) $$p-\hat{p}=\frac{P_{xx}}{3}-\frac{\langle P_{T}\rangle }{3}$$

The normal pressure is equal to the tangential pressure in the thermodynamic limit (the bulk fluid). For the bulk fluid;

(A6) $$P_{xx}=\langle P_{T}\rangle =p^{f}$$

These relations mean that there is no difference between *p* and $\hat{p}$.

With the assumption used in Equation (A2), $P_{xx}$, is constant in the direction perpendicular to the pore surface, and

(A7) $$P_{xx}=p^{f}$$

also away from the thermodynamic limit. By combining Equation (A2) with the definition of $\hat{p}$, Equation (11), we obtain

(A8) $$\langle P_{T}\rangle =p^{f}-\frac{\gamma^{fs}}{a}$$

By introducing Equations (A7) and (A8) into Equation (A5), we obtain

(A9) $$p-\hat{p}=\frac{p^{f}}{3}-\frac{p^{f}}{3}+\frac{\gamma^{fs}}{3a}$$

which is

(A10) $$p-\hat{p}=\frac{\gamma^{fs}}{3a}$$

The difference between the differential pressure *p* and the integral pressure $\hat{p}$ in this equation is the same as the difference in Equation (16), which was obtained using Hills formalism.
